# A Treatise on Midwifery

**Published:** 1876-10

**Authors:** 


*A Treatise on the Science and Practice of Midwifery.* By W. S. Playfair, M. D., F. R. C. P. Philadelphia: Henry C. Lea, 1876. Buffalo: Theo. Butler & Son.

As Dr. Playfair's work is written mostly for Englishmen, and from an English point of view, there are in it some statements which will not wholly coincide with the views of the majority of American readers. It is not, however, to be considered as a treatise, and is not, therefore, to be viewed as such. It is written in an easy, familiar style, such as will make it agreeable as a text-book to students, and as a convenient work of reference for physicians. The numerous excellent wood-cuts will add much to the simplicity of the text, and will impress the teachings of the author upon the reader. The work is divided into five sections, which consider in turn: The anatomy and physiology of the organs concerned in parturition, pregnancy, labor, obstetric operations, and the puerperal state. The author, unlike some English writers, announces his preference for the long forceps which he very properly states can be used in all cases, and with as much ease and gentleness as the shorter ones. He evidently labors under a misaprehension when he says, that American and Continental forceps are larger and more cumbrous than the English. This fact is largely true in reference to the German and other Continental instruments, the handles of which are heavy and unwieldy, but the American instrument in greatest favor is light and graceful, and possessed of an advantage not found in many European instruments, the second or pelvic curve. The author includes under puerperal septicæmia, septicæmia, pyæmia and puerperal fever. He seems to think the view which holds that puerperal fever is something separate and distinct is a mistaken one, that it is identical with what surgeons have known as septicæmia and pyæmia. While we acknowledge some force in the arguments for this side of the question we are still inclined to look upon puerperal fever as a disease special and incident to the puerperal state. This is as we understand it the position taken by Prof. Barker in his excellent work, with which our readers are many of them familiar. The general style of the work, the print, etc , are of a first class order.

